# On-target versus off-target effects of drugs inhibiting the replication of SARS-CoV-2

**DOI:** 10.1038/s41419-020-02842-x

**Published:** 2020-08-19

**Authors:** Allan Sauvat, Fabiola Ciccosanti, Francesca Colavita, Martina Di Rienzo, Concetta Castilletti, Maria Rosaria Capobianchi, Oliver Kepp, Laurence Zitvogel, Gian Maria Fimia, Mauro Piacentini, Guido Kroemer

**Affiliations:** 1 Equipe labellisée par la Ligue contre le cancer, Université de Paris, Sorbonne Université, INSERM UMR1138, Centre de Recherche des Cordeliers, Paris, France; 2 Metabolomics and Cell Biology Platforms, Gustave Roussy, Villejuif, France; 3 Department of Epidemiology, Preclinical Research, and Advanced Diagnostics, National Institute for Infectious Diseases ‘L. Spallanzani’ IRCCS, Rome, Italy; 4 Department of Molecular Medicine, Sapienza University of Rome, Rome, Italy; 5 Department of Biology, University of Rome ‘Tor Vergata’, Rome, Italy; 6 Gustave Roussy Comprehensive Cancer Institute, Villejuif, France; 7 INSERM U1015, Villejuif, France; 8 Center of Clinical Investigations in Biotherapies of Cancer (CICBT), 1428 Villejuif, France; 9 Faculty of Medicine, Université Paris Saclay, Le Kremlin-Bicêtre, France; 10 Université Paris Sud, Paris Saclay, Faculty of Medicine, Kremlin Bicêtre, France; 11 Suzhou Institute for Systems Medicine, Chinese Academy of Medical Sciences, Suzhou, China; 12 Pôle de Biologie, Hôpital Européen Georges Pompidou, AP-HP, Paris, France; 13 Department of Women’s and Children’s Health, Karolinska Institutet, Karolinska University Hospital, Stockholm, Sweden

**Keywords:** Virtual screening, Viral infection

## Abstract

The current epidemic of coronavirus disease-19 (COVID-19) caused by severe acute respiratory syndrome coronavirus-2 (SARS-CoV-2) calls for the development of inhibitors of viral replication. Here, we performed a bioinformatic analysis of published and purported SARS-CoV-2 antivirals including imatinib mesylate that we found to suppress SARS-CoV-2 replication on Vero E6 cells and that, according to the published literature on other coronaviruses is likely to act on-target, as a tyrosine kinase inhibitor. We identified a cluster of SARS-CoV-2 antivirals with characteristics of lysosomotropic agents, meaning that they are lipophilic weak bases capable of penetrating into cells. These agents include cepharentine, chloroquine, chlorpromazine, clemastine, cloperastine, emetine, hydroxychloroquine, haloperidol, ML240, PB28, ponatinib, siramesine, and zotatifin (eFT226) all of which are likely to inhibit SARS-CoV-2 replication by non-specific (off-target) effects, meaning that they probably do not act on their ‘official’ pharmacological targets, but rather interfere with viral replication through non-specific effects on acidophilic organelles including autophagosomes, endosomes, and lysosomes. Imatinib mesylate did not fall into this cluster. In conclusion, we propose a tentative classification of SARS-CoV-2 antivirals into specific (on-target) versus non-specific (off-target) agents based on their physicochemical characteristics.

## Introduction

Contrasting with endemic coronaviruses (CoVs) that account for a rather small percentage of lethal respiratory infections, a few *betacoronaviruses* are both highly pathogenic and transmissible, as this has been documented for Middle East respiratory syndrome (MERS)-CoV, severe acute respiratory syndrome (SARS)-CoV, and the new SARS-CoV-2. This latter virus is causing a pandemic that started in 2019 and hence receives the name coronavirus disease-19 (COVID-19). Thus far, no efficient treatment of COVID-19 has been developed, spurring interest in the identification of pharmacological agents that block viral infection or replication^1^.

A recently published pharmacological screen identified 66 druggable human proteins or host factors targeted by 69 compounds (among which 29 were FDA-approved drugs, 12 drugs in clinical trials, and 28 preclinical compounds) with SARS-CoV-2 antiviral activity. Some among these agents were indeed able to inhibit SARS-CoV-2 replication in African green monkey kidney epithelial Vero E6 cells, as determined by two cooperating Institutions, Mount Sinai Hospital (New York, USA) and Pasteur Institute (Paris, France), using low-throughput assays based on the immunofluorescence-based detection of viral proteins and the levels of RT-PCR-detectable viral RNA, respectively^2^.

Based on this information, publicly available information in databases (in particular PubChem), sophisticated cheminformatics tools, as well as on the published literature, we performed a critical analysis of the aforementioned dataset to distinguish agents that are likely to act off-target (as they are lysosomotropic, based on their physicochemical characteristics: weak bases with lipophilic properties that accumulate in acidic vesicles including lysosomes) from agents that might act on-target. Of note, we identified imatinib mesylate, a tyrosine kinase inhibitor, as a new putative anti-COVID-19 agent.

## Material and methods

### General statistical procedures

Unless explicitly mentioned, all statistical evaluations were performed using the R software (https://www.r-project.org/). **“**Wet” experiments were performed three times yielding similar results. The replicate with the lowest variation in controls was selected for statistical evaluation by means of a paired two-sided Student’s *t*-test (with Welch correction for compensating unequal variances), assuming that the samples distribution was Gaussian. Molecular descriptor correlations were evaluated using the Pearson method (searching for linear relationships between variables); group comparisons were performed by means of a non-parametric Mann–Whitney test, as most descriptors were noticeably not following a normal distribution. Datasets were summarized in Supplemental Tables 1 and 2.

### Datasets of molecular descriptors

Most descriptors were computed based on Simplified Molecular Input Line Entry Specification (SMILES) from each compound using the Chemistry Development Kit (CDK, https://cdk.github.io/) and its R wrapper package *rcdk*^3^. Additional descriptors (protonation and solvent-partitioning properties) were calculated using the dedicated plugins from ChemAxon software (https://chemaxon.com/products/calculators-and-predictors). SMILES were retrieved either directly from the reference paper^2^ or from the PubChem database (https://pubchem.ncbi.nlm.nih.gov/) using the PUG-REST API. SMILES of ketoamide inhibitors were generated from their planar formula using the ChemAxon MarvinSketch tool (https://chemaxon.com/products/marvin).

### Fingerprint-based drug classification

Molecular fingerprints (as described in the Pubchem repository) were generated from SMILES using the R *ChemmineR* package^4^. Pairwise compound similarities (ranging from 0 to 1) were thereafter calculated to generate a distance matrix and finally transformed into a dendrogram allowing for the identification of drug clusters.

### Hit classification based on protonation properties

A total of 13 descriptors was computed using the *cxcalc* function from ChemAxon, including the isoelectric point (pI, pH where the molecule charge is globally neutral), pKa of ionizable groups (thereafter averaged to generate the pH at equilibrium, pHe), the average molecule charge, logP (Solvent-partitioning coefficient for neutral species), logD (Solvent-partitioning coefficient for charged species), the hydrophilic-lipophilic balance number (HLB), and the polar surface area (PSA). When pH-dependent, these parameters were calculated at both pH4.5 and pH7.4, corresponding to lysosomal and physiological environments respectively, and subtracted one from the other, leading to dCharge, dLogP and dLogD values. From these 13 descriptors, five were retained from the best separating principal component analysis (PCA). In more detail, different parameter subsets were randomly selected and submitted to a PCA using the *FactoMineR* R package, and the resulting three main components were used to visually cluster compounds; an optimal choice was performed when the cited components were clearly separating hits into distinct groups. These parameters were thereafter used for separating precisely hits in two groups based on hierarchical clustering.

### Training set curation and preparation

A raw primary data table of 75 entries (from Bojkova et al.^2^) and 291 parameters was generated using the methods described above. Parameters with more than 75% of missing entries, null standard deviation, or highly correlated to others (Pearson|R|>0.9) were removed from the dataset, to generate a curated matrix with 112 valid parameters (Supplemental Table 2). Each column from the matrix was finally centered and scaled to variance unit.

### Determination of most discriminating descriptors

First, lyso-like or other hits were compared with background for each valid descriptor by means of a Mann–Whitney test. From there, seven lyso-like specific descriptors were selected by setting an arbitrary threshold for the *p*-value to 0.0125 in both cases. These selected descriptors were thereafter used for training a random forest classification model using the R *caret* package. This machine learning tool allowed to classify the relative importance of descriptors for distinguishing drug categories (here classified in a binary fashion as «lyso-like hits» and «other compounds»), by computing the mean decrease of the Gini index (an entropy-like measure of the impurity) over the random forest nodes that were split on them. Of note, class imbalance (one class being undersampled with 9 entries, and the other oversampled with 66) was corrected using the Synthetic Minority Oversampling Technique (SMOTE).

### Prediction of putative hit reliability

A manual selection of 39 drugs described for their inhibitory effect on SARS-CoV-2 replication (among which 20 had strong documented proofs) was made for further investigation (Supplemental Table 1). First, a raw descriptor test dataset was generated as described above; columns were then validated, centered, and scaled to variance unit using the exact same procedures that used for training set. The obtained data set was used as an input for the previously trained random forest model to classify compounds into lyso-like hits or background.

### Cell culture and treatments

Vero E6 cells (ATCC® Number CRL-1586™) were maintained in Modified Eagle Medium (MEM, Sigma Aldrich) supplemented with 10% heat inactivated fetal bovine serum (FBS, Invitrogen) at 37 °C in a humidified atmosphere of 5% CO_2_. Vero E6 cells were exposed to SARS-CoV-2 isolate (2019-nCoV/Italy-INMI1, available from EVAg, Ref-SKU: 008V-03893) for 1 h at 37 °C at a multiplicity of infection (MOI) of 0.01. At the end of the adsorption period, cells were washed and, where indicated, treated with imatinib mesylate (Sigma Aldrich) 10 μM at the end of the adsorption period. Treatment was repeated after 24 h. At 24 and 48 h post infection, cells were harvested and assayed for SARS-CoV-2 RNA and protein content.

### Viral RNA analysis

Viral RNA was extracted from Vero E6 cells using Trizol (Life Technologies), according to the manufacturer’s instructions. SARS-CoV-2 RNA was amplified by real-time quantitative RT-PCR (qRT-PCR) in Rotor-GeneQ Real-Time cycler (Qiagen) using RealStar® SARS-CoV-2 RT-PCR Kit 1.0 (Altona Diagnostic GmbH). A standard curve prepared through serial dilutions of the EURM-019 single-stranded RNA (ssRNA) fragments of SARS-CoV-2 (https://crm.jrc.ec.europa.eu/p/EURM-019) was used to determine the viral load. Levels of viral RNAs were normalized to the housekeeping gene L34 level using the equation 2-ΔCt.

### Viral protein analysis

Immunoblotting was performed lysing cells in Tris buffer (10 mM Tris pH 8.0, 150 mM NaCl, 10% glycerol, and 1% Triton-X100) complemented with protease and phosphatase inhibitors (Protease inhibitor cocktail plus, 5 mM sodium fluoride, 0.5 mM sodium orthovanadate, 1 mM sodium molibdate, and 0.5 mM PMSF (all reagents were from Sigma-Aldrich)). Proteins were separated on SDS PAGE gels (Bio-Rad) and electroblotted onto nitrocellulose (Whatman Amersham) or PVDF (Millipore) membranes. The primary antibodies used in this study were anti-SARS-CoV Nucleocapsid (200–401-A50 Rockland Immunochemicals), anti-SARS-CoV-2 NSP8 [5A10] (GTX632696 GENETEX), anti-SARS-CoV-2 ORF7a [3C9] (GTX632602, GENETEX), PARP antibody (BK9542S, Cell Signaling), and anti-HSP90 alpha/beta (F-8) (sc-13119, Santa Cruz Biotech). Detection was achieved using horseradish peroxidase-conjugate secondary antibodies (Jackson ImmunoResearch Laboratories) and ECL (Immobilon, Millipore). Signals were acquired using a ChemiDoc Imaging System (Bio-Rad).

## Results

### Selected physicochemical parameters correlate with SARS-CoV-2 antiviral activity

**Fig. 1:**
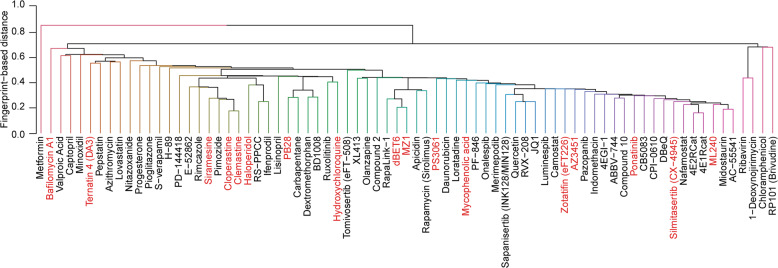
Classification of putative SARS-CoV-2 replication inhibitors. A hierarchical clustering was applied to the distance matrix computed using compounds pairwise fingerprint similarity calculations. The branches of the dendrogram are colored based on a cut-off distance of 0.15. Compounds in red correspond to hits identified in Paris and/or New York.

We used the information from a screen that identified agents able to inhibit the replication of SARS-CoV-2 in non-human primate cells^2^ to identify possible common structural features (based on their planar configuration) explaining such effects. Indeed, the published screen, which was based on a selection of agents that had reported effects on proteins that might be involved in SARS-CoV-2 activation, only confirmed a significant antiviral effect for a fraction among them, yielding 17 ‘hits’ out of 75 agents with known physicochemical characteristics^2^. Of note, PubChem Structure Fingerprint-based analysis classified these agents into rather distinct groups, hence failing to reveal a general pattern of characteristics, though with the exception of a few pairs (such as cloperastine + clemastine, dBET6+MZ1, Zofatifin + AZ3451). Rather, the hit compounds were scattered all over the dendrogram. Hence, this fingerprint-based analysis failed to distinguish active and inactive compounds (Fig. 1).

**Fig. 2:**
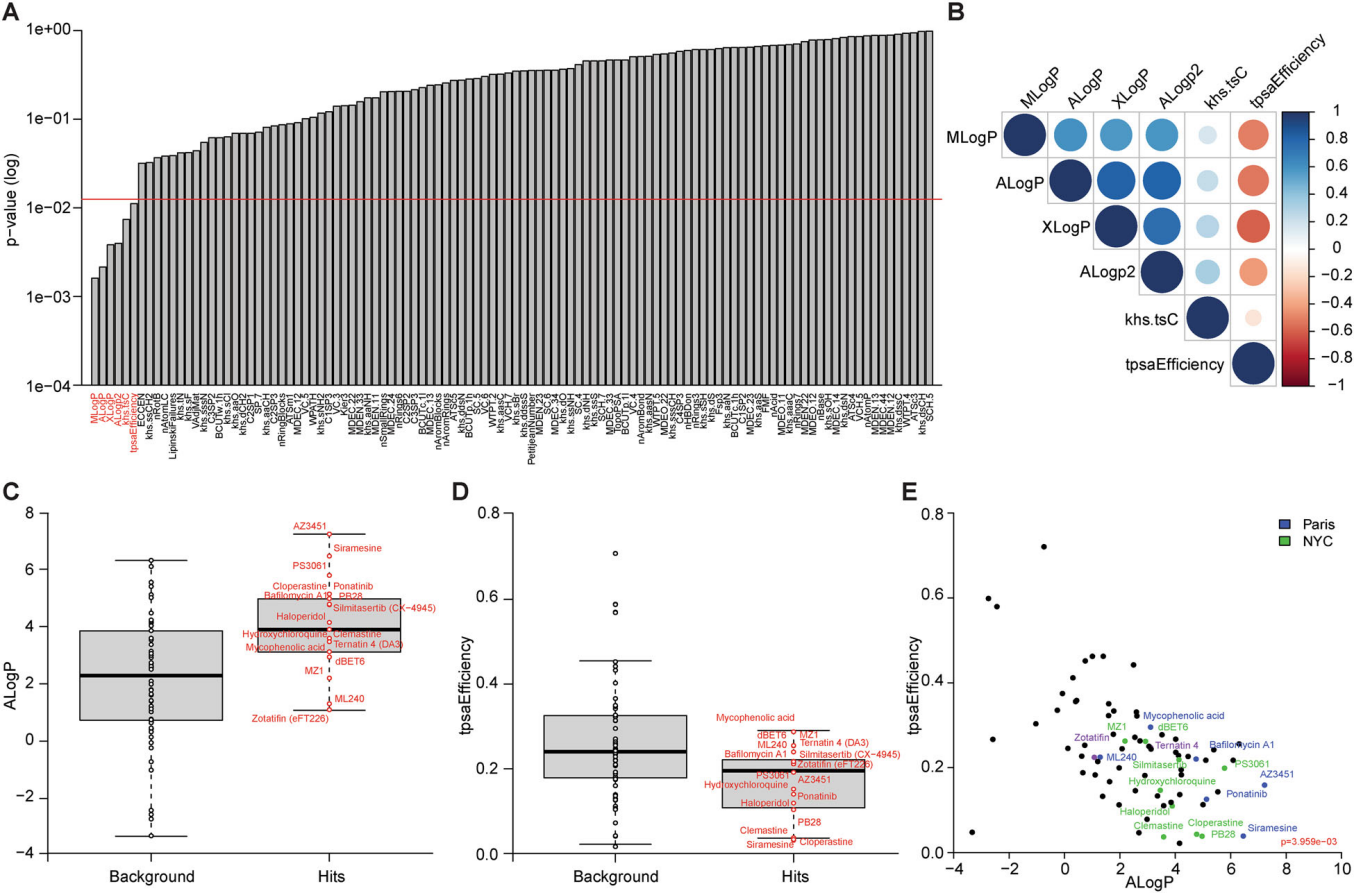
Determination of discriminating chemical descriptors. **a** Molecular descriptors were computed by using the CDK library and were compared individually between hit and background groups by means of a Mann–Whitney test. The obtained *p*-values are ranked and reported in a barchart. Descriptors with a *p*-value < 0.0125 are depicted in red, and were submitted to a correlation analysis. The resulting correlation matrix indicating Pearson’s *R* coefficients is shown in **b**. **c**–**e** Two of the relevant descriptors with lowest intra-group deviations were selected. Their distributions into hits or background are depicted as boxplots (**c**, **d**) or reported as a bi-parametric dot plot (**e**). A 2-D statistical test was performed by means of a Hotelling test; the resulting *p*-value is reported in red.

For this reason, we decided to study more complex and variate quantitative molecular descriptors to unravel a potential structure-function relationship. We calculated for each of the 75 studied compounds a set of 108 molecular descriptors (computed using the CDK API) that were then tested statistically for median differences between the hit and the background groups. Six physicochemical characteristics were significantly different (Mann–Whitney test *p* < 0.0125) among active and inactive compounds (Fig. 2a). Four among these six physicochemical characteristics (MLogP, AlogP, XLogP, and ALogp2), which all reflect lipophilicity (the higher, the more lipophilic), strongly correlated among each other. However, these four parameters all anti-correlated with topological polar surface area (TPSA) efficiency, which reflects the ability of a drug to penetrate into a cell (the lower, the higher the ability to penetrate; Fig. 2b). Indeed, the active, SARS-CoV-2-inhibitory agents (‘hits’) tended to have a higher lipophilicity (Fig. 2c) and a lower TPSA (Fig. 2d) than the inactive compounds. These effects were significantly different between active and inactive compounds (with a combined *p*-value of 0.004 in a 2-D Hotelling test using AlogP and TPSA) and did not distinguish between the hits identified at Mount Sinai Hospital, NY, and at the Pasteur Institute, Paris. However, there was still a major overlap between the active and the inactive compounds (Fig. 2e), meaning that the classifier required further improvement.

### Lysosomotropic versus non-lysosomotropic SARS-CoV-2 antiviral drugs

**Fig. 3:**
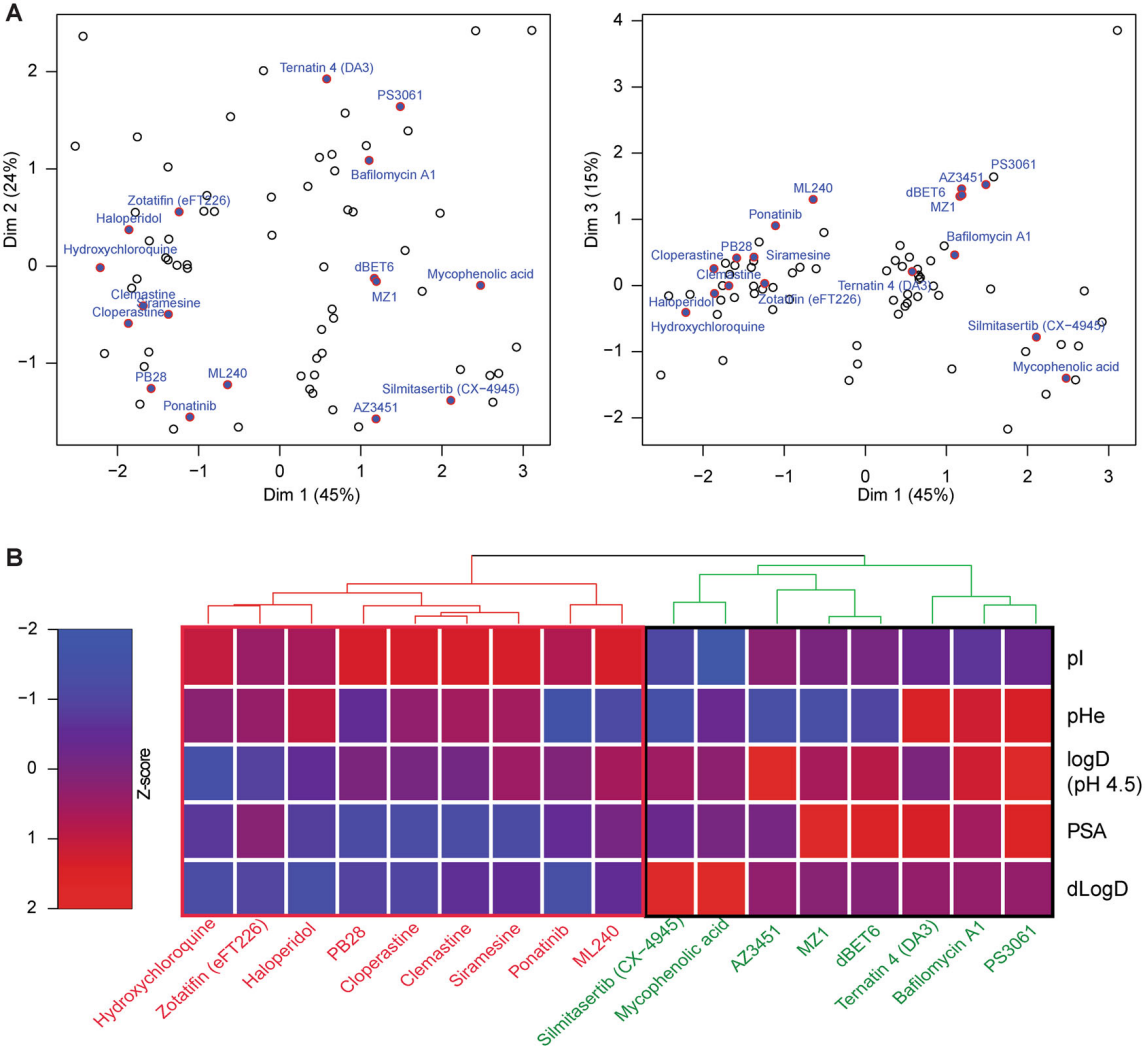
Hits clusterization based on their protonation properties. Several protonation as well as solvent-partitioning properties were computed for each of the 75 compounds from the original set using ChemAxon software, and submitted to a principal component analysis. **a** Data projections on the resulting three main dimensions are represented as dot plots. Hits are indicated in blue. **b** The most important parameters from the main components are reported for hits as a heatmap. The two groups resulting from subsequent hierarchical clustering are reported in red and blue colors, respectively.

There has been considerable controversy about the effects of hydroxychloroquine, which inhibits SARS-CoV-2 replication in vitro^5^, but fails clinically to clear SARS-CoV-2 and to improve Covid-2, at least in hospitalized patients^6–9^. Hydroxychloroquine is a prototypic lysosomotropic agent, meaning that, due to its lipophilicity (at neutral pH) and low TPSA, it attains the cytoplasm and then enriches in the acidic environment of lysosomes because it is protonated and hence trapped within the lysosomal lumen. Due to its selective enrichment in this organelle, it perturbs lysosomal functions including autophagy and may even cause lysosomal membrane permeabilization (LMP)^10,11^. Driven by this consideration, we computed 13 chemical descriptors linked to protonation including the isoelectric point and used them to investigate their impact on the classification of SARS-CoV-2 drugs by principal component analysis. Although these parameters did not entirely discriminate hits from non-hits, they did separate the hits into two clearly distinct groups (Fig. 3a). We then retained the five most important protonation-relevant physicochemical parameters for further analysis: pI (the pH at which the molecule charge is globally neutral), pHe (pH at equilibrium, the average pKA of ionizable groups), logD (solvent-partitioning coefficient for charged species), PSA (polar surface area), and dlogD (difference of solvent-partitioning coefficient for charged species, calculated by subtracting the values at pH4.5 and pH7.4, corresponding to the lysosomal and cytosolic pH, respectively). These five parameters allowed to separate the hits from the screen into two groups that we considered as lysosomotropic (with hydroxychloroquine as a representative compound) and non-lysosomotropic (Fig. 3b).

**Fig. 4:**
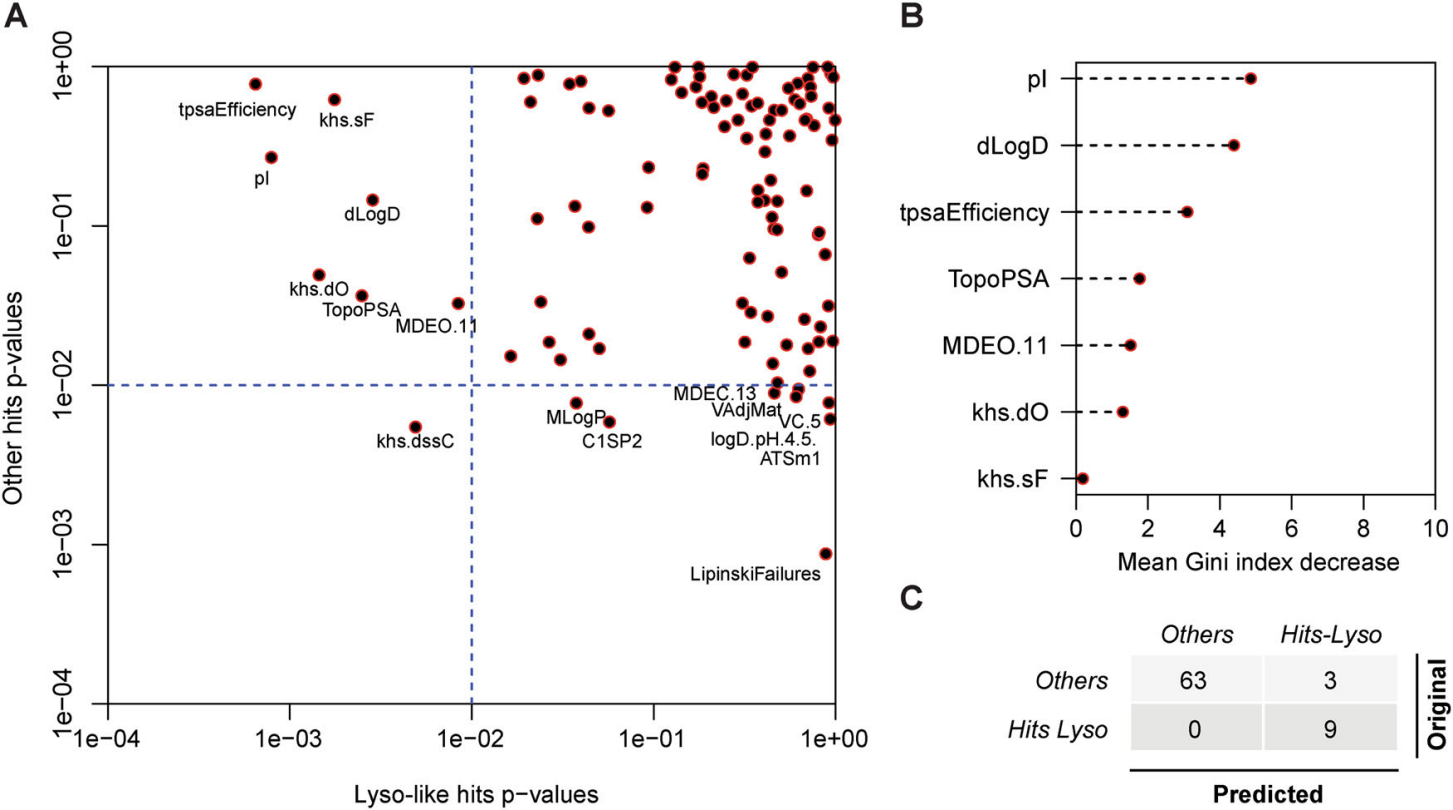
Determination of discriminating chemical descriptors. **a** Molecular descriptors were computed by using the the CDK library and ChemAxon software, and each of them was compared between hit and background groups by means of a Mann–Whitney test. The obtained *p*-values for either lyso-like or other hits are reported in a dot plot. Thresholding values (*p* = 0.0125) are indicated as blue dashed lines. **b**, **c** A random forest classification model was built using lyso-like specific descriptors as a predicting tool. The variables importancy (as the mean decrease of the Gini index) for building the model is reported in a dot plot (**b**), the confusion matrix (indicating model accuracy) is depicted in **c**.

We then investigated the possibility to accurately classify the compounds based on their molecular descriptors. First, we computed the *p*-values (Mann–Whitney test) that discriminate lysosomotropic hits (as listed in Fig. 3b) from non-hits versus the *p*-values that discriminated non-lysosomotropic hits from non-hits. Non-lysosomotropic hits differed from non-hits mostly based on ‘Lipinski failures’, a composite index predicting whether a small molecule may be useful as a drug^12^. Lysosomotropic hits differed from non-hits, mostly based on tpsaEfficiency, khs.sF, pI, and dLogD (Fig. 4a). Next, we used a random-forest machine learning approach, which is able to build a prediction model with low overfitting risk, while indicating the importance of explicative variables thanks to the Gini impurity index (Fig. 4b). When such an approach was used to classify compounds into ‘lysosomotropic hits’ versus ‘other’ compounds^2^, it resulted in the accurate classification of all 9 ‘lysomotropic hits’ (error rate 0%), as well as in the accurate classification of most (63 out of 66) ‘other’ agents (error rate 4.5%), as shown in the confusion matrix (Fig. 4c). Three variables were particularly important for discriminating groups, namely pI, dLogD, and tpsaEfficiency (Fig. 4b). In short, lysosomotropic agents with anti-SARS-CoV-2 activity can be accurately identified by their physicochemical properties.

### A classification of putative anti-SARS-CoV-2 drugs including imatinib mesylate

As three compound descriptors (pI, dLogD, and tpsaEfficiency) were intimately linked to the biological activity of SARS-CoV-2-inhibitory lysosomotropic agents, we decided to investigate their status among 39 other drugs, chosen for their documented capacity to modulate the SARS-CoV-2 effect. Thus, we used the insights obtained above to classify agents that have been published to inhibit SARS-CoV-2 replication such as three α-ketoamide inhibitors^13^, abiratone^14^, azithromycin^15^, bexarotene^14^, β-d-N4-hydroxycytidine (NHC; EIDD-1931)^16^, camostat mesylate^17^, cepharanthine^18^, cetilistat^14^, chloroquine^19^, chlorpromazine:^20^, diiodohydroxyquinoline^14^, emetine^21^, homoharringtonine (also called omacetaxine mepesuccinate)^21,22^, ivermectin^23^; lopinavir^21^, mefloquine^18^, remdesivir^21^, and selamectin^18^.

**Fig. 5:**
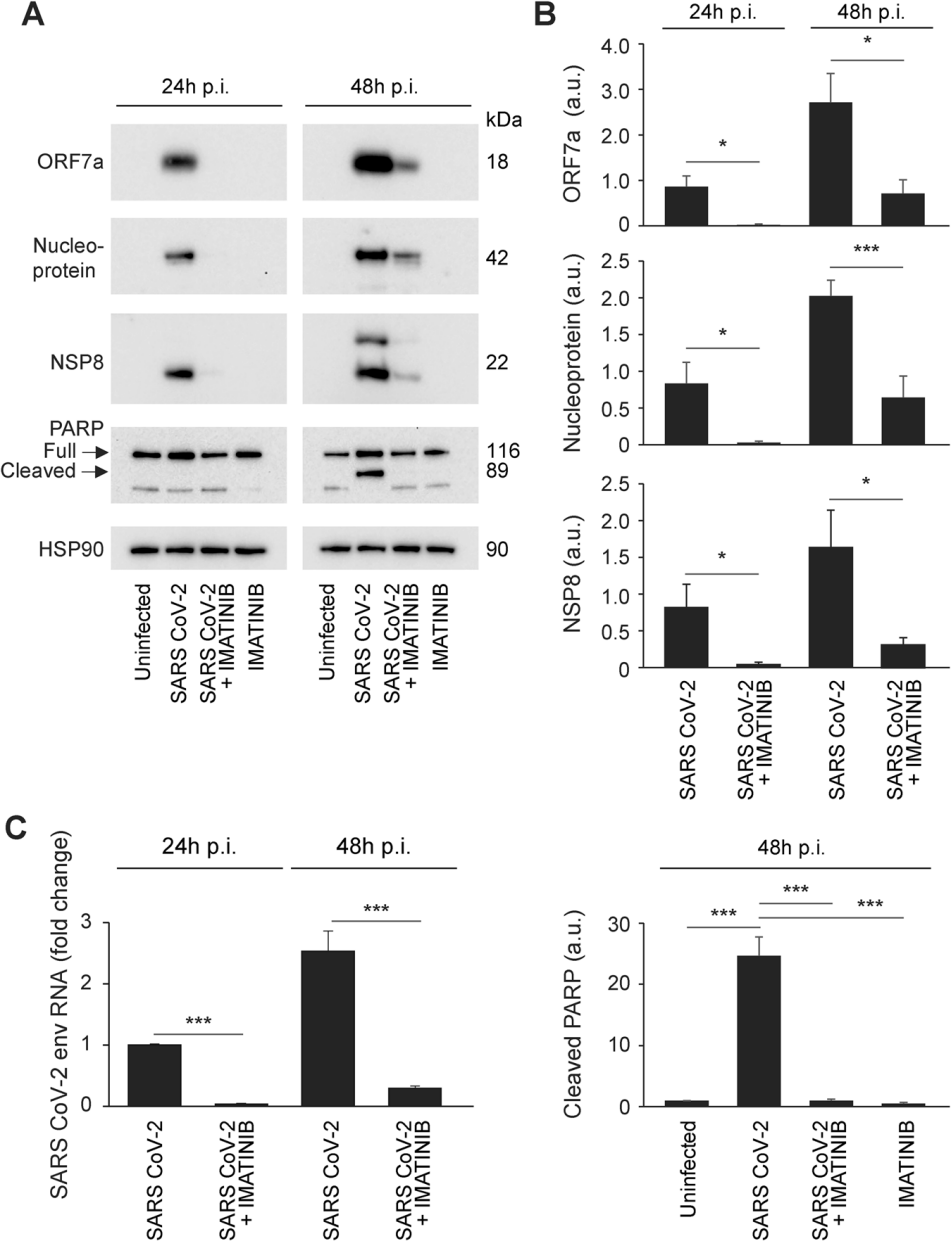
Inhibition of SARS-CoV-2 infection by imatinib mesylate in Vero E6 cells. Vero E6 cells were infected with the SARS-CoV-2 isolate (2019-nCoV/Italy-INMI1) for 1 h at 37 °C at a multiplicity of infection (MOI) of 0.01. At the end of the adsorption period, cells were treated with imatinib mesylate 10 mM or left untreated. Treatment was repeated after 24 h. At 24 and 48 h post infection, cells were harvested and assayed for SARS-CoV-2 intracellular protein (**a**, **b**) and RNA (**c**) levels by immunoblotting and RT-qPCR, respectively. In addition, PARP cleavage was monitored by immunoblotting to evaluate the level of cell death in infected cells (**a**, **b** lower panel). Representative images of immunoblotting results are shown in **a**; normalized quantification and statistical analysis of immunoblotting data from three experiments are described in **b** (a.u.: arbitrary unit). Viral RNA levels are reported as fold changes with respect to the amount detected at 24 h post infection (p.i.). Data represent means ± SD from triplicates. *p < 0.05; ***p < 0.001; paired Student’s *t* test.

Imatinib mesylate, a clinically used inhibitor of the receptor tyrosine kinase c-Abl (encoded by the ABL1 gene) that also inhibits ABL2 (an intracellular paralog of ABL1)^24^, has been reported to inhibit the replication of MERS-CoV and SARS-CoV, likely by an on-target effect, because knockdown of ABL2 (but not ABL1) also inhibits MERS-CoV and SARS-CoV^25^. In our hands, imatinib mesylate also suppressed the replication of SARS-CoV-2 in Vero E6 cells (Fig. 5), prompting us to include this agent in the list of anti-SARS-CoV-2 agents that we completed with agents that inhibit SARS-CoV-2 according to internet communications by biotechnology companies and the pharmaceutical industry.

**Fig. 6:**
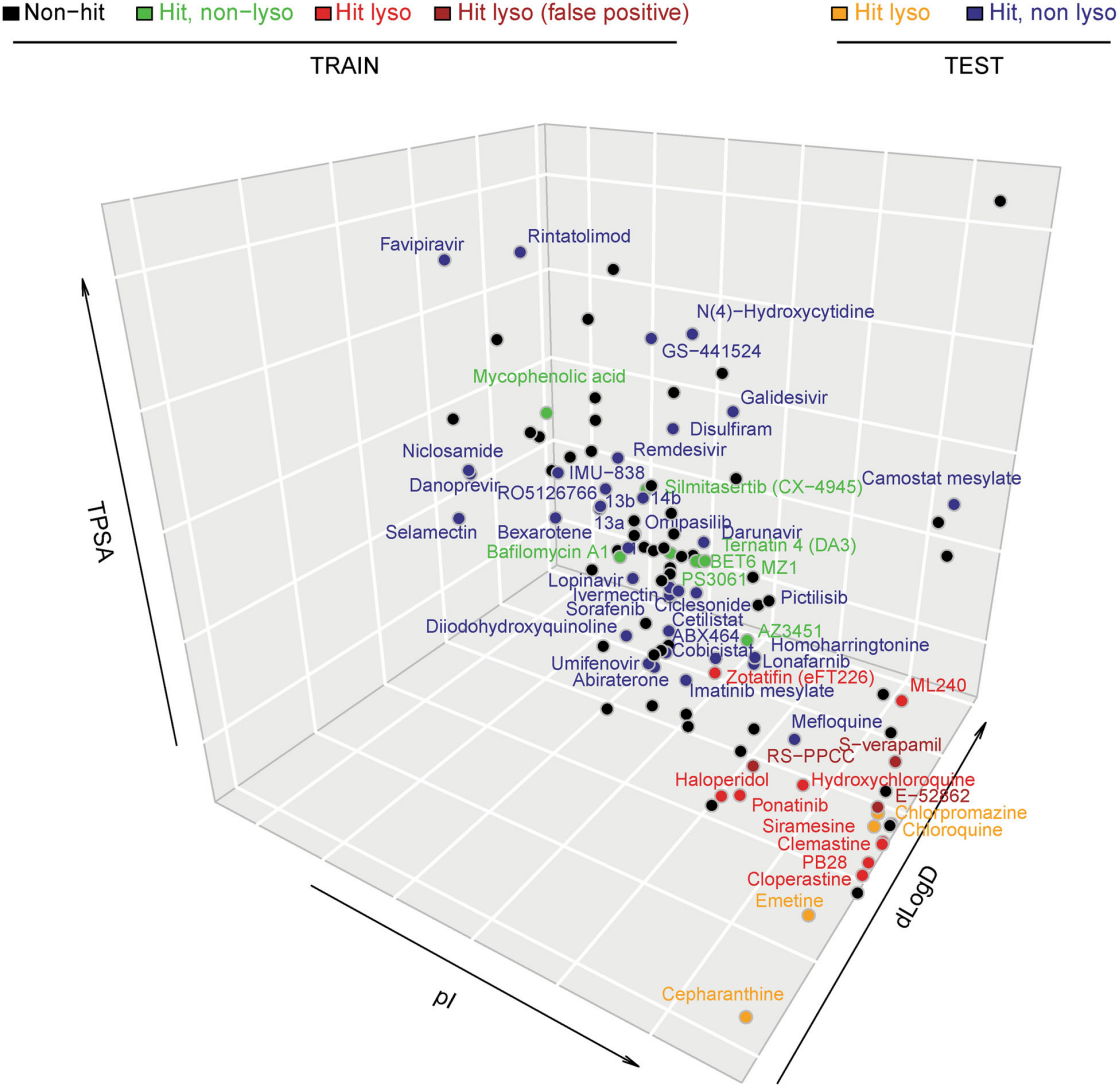
Machine-learning-based enrichment in putative hits. A trained random forest classification model was used to predict the potential antiviral effect of 39 active new compounds described in the literature. The three most important parameters that allowed to build the prediction model were plotted in a 3D scatterplot for both the original 75 drugs (training set) and these last compounds (test set). Prediction results and model accuracy are represented by colors, as indicated in the legend.

Random forest-based classification of the 114 drugs (performed based on the algorithm used in Fig. 4b, c), identified a cluster of agents with reported anti-SARS-CoV-2 activity that included cephararanthine, chloroquine, chlorpromazine, and emetine. When projected on the three most important descriptors of the classifier (pI, dLogD, and tpsaEfficiency), mefloquine clustered in proximity of these compounds even if not classified as “hit” by the AI model. In sharp contrast, imatinib mesylate did not fall into this cluster (Fig. 6).

## Discussion

COVID-19 poses a major challenge to public and private health providers because there is no effective cure for the disease. Drugs that are currently in clinical evaluation have been selected based on their capacity to inhibit SARS-CoV-2 replication in vitro or to dampen excessive inflammatory reactions in response to SARS-CoV-2 infection. Here, we centered our attention on agents that have a potential antiviral effect while analyzing their molecular properties. We identified a cluster of agents with a high isoelectric point (pI), a low dLogD (meaning that they get protonated and their hydrophilicity increases at a pH of 4.5) and tpsaEfficiency (meaning that they easily penetrate membranes) that appear to act as lysosomotropic agents^26^.

Among these lysosomotropic agents, chloroquine and hydroxychloroquine (which are used as antimalarial agents, as well as for the treatment of rheumatoid arthritis and systemic lupus erythematosus) are prototypic lysosomotropic and autophagy-inhibitory drugs, although they can mediate part of their cytotoxic activity through autophagy-independent (but mitochondrion-dependent) effects^10,27,28^. The antihistaminergic clemastine affects autophagic flux in mice bearing the SOD1 G93A mutation while reducing motoneuron loss in this model of amyotrophic lateral sclerosis^29^. Fluorescent derivatives of the σ receptor ligand 1-cyclohexyl-4-[3-(5-methoxy-1,2,3,4-tetrahydronaphthalen-1-yl)propyl]piperazine (PB28) have been shown to enrich in lysosomes^30^. Siramesine, a σ2 receptor ligand similarly is a lysosomotropic and LMP-inducing agent^31^, although it may also target mitochondria^32^. Haloperidol, yet another σ2 receptor ligand, can cause lysosomal alkalinisation^33^ and inhibit autophagy^34^. Chlorpromazine, an antipsychotic, is yet another drug with a well-established lysosomotropism that modulates autophagy^35,36^ and inhibits clathrin-mediated endocytosis^37^. Cepharanthine and emetine also reportedly inhibit autophagy in vitro and in vivo, respectively^38,39^.

Among these lysomotropic agents, chloroquine and (more so) hydroxychloroquine have been massively tested for the treatment of COVID-19, alone or in combination with macrolide antibiotics, yielding some encouraging results in uncontrolled observational studies^40,41^, but ultimately failing in randomized trials^6–8^, and even preclinical experimentation in ferrets^42^ and non-human primates (https://www.researchsquare.com/article/rs-27223/v1). We suspect that the other agents classified among lysosomotropic agents that are currently undergoing clinical evaluation such as chlorpromazine (NCT04366739) or mefloquine (NCT04347031), will similarly fail due to their non-specific (i.e. off-target) mode of action that is based on their lysosomotropism. It should be noted that this tropism for lysosomes (and other acidic organelles) might also explain the QT prolongation that underlies drug-induced arrhythmia, because several (but not all) of the agents falling into this class are well known for this potentially lethal (but rare) side effect, as documented for chlorpromazine, chloroquine, haloperidol, and hydroxychloroquine^43–45^.

In contrast to the aforementioned lysosomotropic agents, a few others may mediate their effects through specific mechanisms. This applies for example to remdesivir, which reduces viral replication in COVID-19 patients, coupled to a rather modest clinical improvement^46,47^. However, remdesivir must be administered intravenously, has major side effects and is a recently developed, expensive drug. Oral treatment of COVID-19 with imatinib (which is now off-patent and hence available as a generic at a reduced cost) is being evaluated in clinical trials in France (NCT04356495 and NCT04357613), the Netherlands (EudrCT: 2020-001236-10), Spain (NCT04346147), and the US, at the University of Baltimore (NCT04394416). Indeed, imatinib is the sole agent that, to our knowledge, inhibits the replication of CoVs through an on-target effect, namely the inhibition of ABL2, as this has been shown for MERS-CoV and SARS-CoV^25,48^. Imatinib has few side effects, as demonstrated by its use for the long-term treatment of chronic myeloid leukemia and gastrointestinal stromal tumors^49,50^. Moreover, this agent has marked immunomodulatory effects in the sense that it reduces inflammation, yet stimulates T and NK responses^51^, supporting its potential utility for the treatment of COVID-19. However, it remains to be seen whether there will be enough cases of COVID-19 to rapidly test imatinib for its clinical utility in Western Europe or in Maryland or whether such trials should be rather attempted in areas in which COVID-19 is rapidly expanding such as the Americas.

In summary, the present work provides a tentative classification of drugs that inhibit SARS-CoV-2 replication into agents that are likely to fail in the clinics due to their high degree of non-specificity and agents that might have a specific mode of action. A whole series of non-specific agents probably inhibit viral replication due to their physicochemical (lysosomotropic) properties leading to a perturbation of acidic organelles rather than due to their specific mode of action (such as blockade of histamine or sigma receptors or the inhibition of tyrosine kinases). Thus, the mode of action of lysosomotropic agents with respect to SARS-CoV-2 replication would be “off-target”. It remains to be determined which among the other, non-lysosomotropic agents may ultimately mediate effects that are specific enough to be considered “on-target”, meaning that they can be described at doses that have been previously established for their clinical use and that yield no or acceptable side effects. Undoubtedly, some of the agents that have been classified as ‘non-lysosomotropic’ still might act on lysosomes, as this is the case for bafilomycin A1, which acts as an inhibitor of the vacuolar-type H^+^ -ATPase (V-ATPase) enzyme and therefore abolishes lysosomal acidification through an on-target effect. Hence, the conjecture that at least some of the ‘non-lysosomotropic’ agents act through non-lysosomal mechanisms will require mechanistic validation by in-depth experimentation, as well as further detailed knowledge on the replication mechanism of SARS-CoV-2.

## Supplementary information

Dataset 1

Dataset 2

Random forest model

Supplemental information
