# Supplementary material for: On-target versus off-target effects of drugs inhibiting the replication of SARS-CoV-2
Source: Cell Death Dis. 2020 Aug 19;11(8):656. doi: 10.1038/s41419-020-02842-x (PMC7434849; doi:10.1038/s41419-020-02842-x)
Supplement: Supplementary file 3 — Random forest model [file 41419_2020_2842_MOESM3_ESM.pdf]

```r
library(caret)

# Test all descriptors
swp = t(sapply(colnames(datr),function(nm){
  c(
    h=wilcox.test(datr[which(cat==3),nm],datr[which(cat==1),nm])$p.value,
    b=wilcox.test(datr[which(cat==2),nm],datr[which(cat==1),nm])$p.value
  )
}))

# Keep relevant descriptors
pt = 0.0125 #p-value threshold
hbp = rownames(swp)[which(swp[,'b']>pt & swp[,'h']<pt)]

# Create binary category
cat2 = cat;cat2[which(cat2==2)]=1

# Supersampling for unbalanced data
sm = SMOTE(cat ~ ., data  = data.frame(cat=as.factor(cat2),datr[,hbp]))

# Create random forest model
set.seed(123)
mod = train(cat~.,data=sm, method='rf',classwt=c(1,5))$finalModel

# Predict original dataset to get confusion matrix
predv = predict(mod,datr[,hbp]);conf=confusionMatrix(data = predv, reference
= as.factor(cat2))
```
