# Supplementary material for: On-target versus off-target effects of drugs inhibiting the replication of SARS-CoV-2
Source: Cell Death Dis. 2020 Aug 19;11(8):656. doi: 10.1038/s41419-020-02842-x (PMC7434849; doi:10.1038/s41419-020-02842-x)
Supplement: Supplementary file 4 — Supplemental information [file 41419_2020_2842_MOESM4_ESM.docx]

**Supplemental information**

**Supp 1. Random forest model**. Piece of R code used for generating the random forest classifier from training dataset & compound category is displayed.

**Dataset 1. Molecular descriptors of the training set.** The molecular descriptors of the 75 drugs constituting the training set were computed by using the CDK library & Chemaxon software and reported in a table.

**Dataset 2. Molecular descriptors of the test set.** The molecular descriptors of the 39 drugs constituting the test set were computed by using the CDK library & Chemaxon software and reported in a table.
